# The Disparity and Dynamics of Social Distancing Behaviors in Japan: Investigation of Mobile Phone Mobility Data

**DOI:** 10.2196/31557

**Published:** 2022-03-22

**Authors:** Zeyu Lyu, Hiroki Takikawa

**Affiliations:** 1 Graduate School Faculty of Arts and Letters Tohoku University Sendai Japan

**Keywords:** COVID-19, social distancing, mobility, time series, tracking, policy

## Abstract

**Background:**

The availability of large-scale and fine-grained aggregated mobility data has allowed researchers to observe the dynamics of social distancing behaviors at high spatial and temporal resolutions. Despite the increasing attention paid to this research agenda, limited studies have focused on the demographic factors related to mobility, and the dynamics of social distancing behaviors have not been fully investigated.

**Objective:**

This study aims to assist in designing and implementing public health policies by exploring how social distancing behaviors varied among various demographic groups over time.

**Methods:**

We combined several data sources, including mobile tracking mobility data and geographical statistics, to estimate the visiting population of entertainment venues across demographic groups, which can be considered the proxy of social distancing behaviors. Next, we used time series analysis methods to investigate how voluntary and policy-induced social distancing behaviors shifted over time across demographic groups.

**Results:**

Our findings demonstrate distinct patterns of social distancing behaviors and their dynamics across age groups. On the one hand, although entertainment venues’ population comprises mainly individuals aged 20-40 years, a more significant proportion of the youth has adopted social distancing behaviors and complied with policy implementations compared to older age groups. From this perspective, the increasing contribution to infections by the youth should be more likely to be attributed to their number rather than their violation of social distancing behaviors. On the other hand, although risk perception and self-restriction recommendations can induce social distancing behaviors, their impact and effectiveness appear to be largely weakened during Japan’s second state of emergency.

**Conclusions:**

This study provides a timely reference for policymakers about the current situation on how different demographic groups adopt social distancing behaviors over time. On the one hand, the age-dependent disparity requires more nuanced and targeted mitigation strategies to increase the intention of elderly individuals to adopt mobility restriction behaviors. On the other hand, considering that the effectiveness of policy implementations requesting social distancing behaviors appears to decline over time, in extreme cases, the government should consider imposing stricter social distancing interventions, as they are necessary to promote social distancing behaviors and mitigate the transmission of COVID-19.

## Introduction

### Background

The rapid global prevalence of COVID-19 has caused an unprecedented public health crisis. Currently, social distancing measures require avoiding unnecessary physical contact and remain the primary public health strategy for mitigating the spread of COVID-19. Several studies have indicated that the transmission rate and death rate seem correlated with how firmly social distancing was implemented [1,2]. However, due to the substantial economic and psychological cost [3,4], social distancing measures are not necessarily accomplished by the whole population. Indeed, previous studies have suggested that protective behaviors are associated with demographic factors, including gender and age [5]. In this sense, variation of infectious cases among demographic groups might be attributed to the varying levels of social distancing behaviors across demographic groups [6-9]. Investigating and understanding the variation in social distancing behaviors across demographic groups can improve the design, implementation, effectiveness, and equity of public health policies, which can reduce the spread of infection and limit the outbreak.

Compliance with social distancing measures during the COVID-19 pandemic has received increasing attention. A primary line of research has conducted surveys to assess individuals’ social distancing behaviors. However, the inherent limitations of surveys determine that they could only capture the condition in a manner limited to relatively coarse areal units or a short-term period. Thus, these studies can only provide insights into 1 point or a short-term situation of social distancing behaviors, and the real-time change in behaviors at higher spatial and temporal resolutions cannot be fully quantified. As the spread of COVID-19 has been due to a long-lasting pandemic, social distancing behaviors should be considered a dynamic process that evolves and shifts in individuals’ perceptions and in policies. Thus, an established mechanism for social distancing behaviors must be examined from a long-term perspective. In this sense, how social distancing behaviors shift in response to different periods remains an open question.

The availability of large-scale and fine-grained aggregated mobility data has allowed researchers to observe the dynamics of social distancing behaviors at high spatial and temporal resolutions [10-12], which can naturally serve as an appropriate assessment to produce a more scalable, long-term analysis. Studies have used mobility data to observe the patterns of human activities during the COVID-19 pandemic and assess the effectiveness of social distancing measures [13-15]. However, most studies have primarily used mobility data to derive coarse information about the estimation of population flow for mathematical simulation and how demographic factors related to mobility have not been fully investigated, as the reliable demographic-specific mobility data remain scarce.

Against these backgrounds, this study explored the social distancing behaviors among various demographic groups over time by using fine-grained mobility tracking data combined with demographic information. More specially, to assess social distancing behaviors by evaluating human mobility data, we focused on the mobility population in entertainment venues. During the current pandemic, officials have strongly encouraged individuals to reduce the frequency of their visits to nonessential leisure establishments, such as restaurants and bars. Notably, an increasing number of infections linked to these settings have been observed, indicating that visiting entertainment venues increases individuals’ risk of infection. Therefore, we considered that visiting entertainment venues is a typical violation of social distancing measures and would be an appropriate proxy for social distancing behaviors.

In addition, this study specified the ﻿voluntary response and policy-induced response to provide a more comprehensive understanding of social distancing behaviors.

First, protection motivation theory suggests that individuals primarily tend to adopt voluntary protective behaviors, including maintaining social distancing, because of their desire to avoid the risk and adverse outcomes of infection [16-18]. Risk perceptions have been important drivers of individuals’ social distancing behaviors during the COVID-19 pandemic. As the perceived susceptibility and perceived severity of the disease can vary across demographic groups [5] and shift over time, it is reasonable to assume there are also demographic differences and time variations in the compliance with social distancing measures.

In addition, public health policy implementation can significantly affect the extent of compliance with social distancing measures. Doubtlessly, beyond the voluntary compliance, policies implemented by governments could accelerate and strengthen compliance with social distancing measures. However, a more nuanced investigation is essential to better understand the impact of public health policy implementation. On the one hand, the effectiveness of the policies is largely dependent on public acceptance and obedience, which might be varied across demographic groups. Therefore, the impact of public health policy on social distancing behaviors can also vary across demographic groups. Governments need to ensure that the policies target all demographic groups, while considering that different demographic groups might not equivalently respond to the policies. On the other hand, ﻿government-initiated interventions have been relatively short lived as their implementation can cause substantial social-economic costs. Notably, the ﻿ resurgence of the epidemic after the lifting of strict social distancing measures may again pose a severe threat and force policymakers to impose stringent social distancing measures repeatedly [19,20]. In this context, the implementation of policy interventions may be enforced multiple times, while the actual effect of these policies on social distancing behaviors during the different phases has not been fully investigated.

In summary, this study aims to address 2 research questions (RQs):

- RQ1: Can an increase in the cases of infection lead to a decrease in the frequency of visits to entertainment venues? Does their relationship vary across demographic groups and different periods of the COVID-19 pandemic?
- RQ2: How does policy intervention affect visits to entertainment venues? Does its impact vary across demographic groups and different periods of the COVID-19 pandemic?

### Research Case

**Figure 1:**
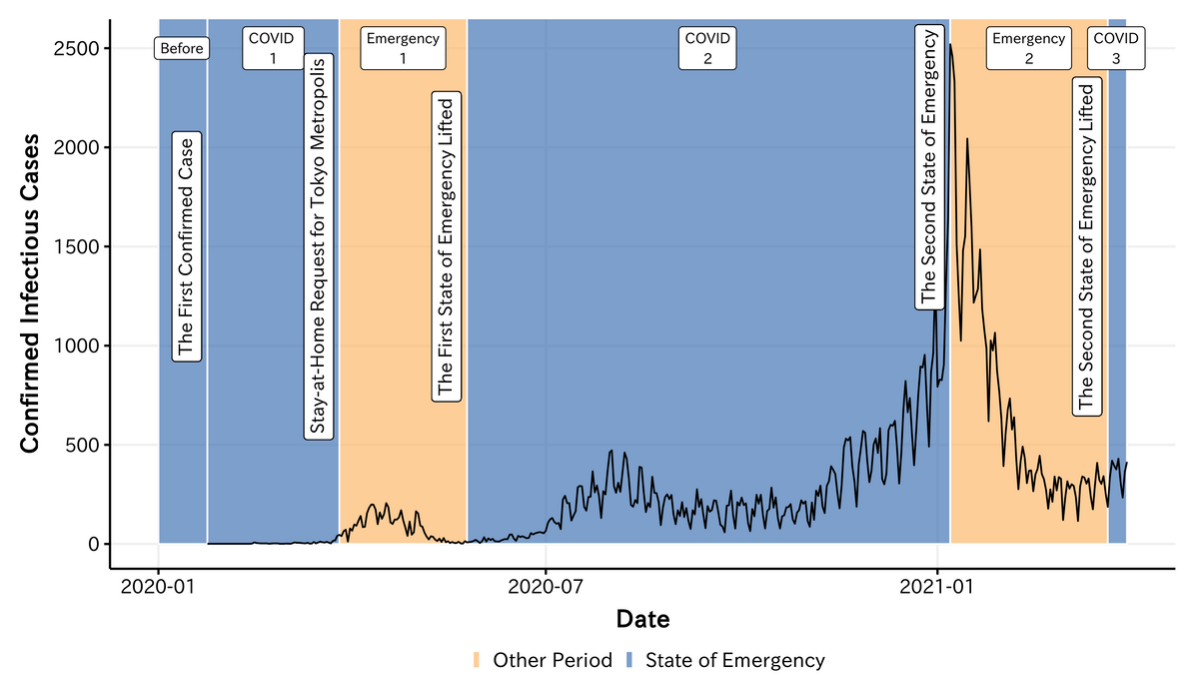
Timeline of COVID-19 prevalence in the Tokyo metropolitan area.

Our focus is on the Tokyo metropolitan area, 1 of the areas most affected by COVID-19. As of March 31, 2021, Tokyo already had 120,986 confirmed COVID-19 cases, which resulted in 1804 deaths. Figure 1 presents the changes in the COVID-19 daily new confirmed cases in Tokyo.

The first case of COVID-19 in Japan was confirmed on January 24, 2020, while confirmed infectious cases remained relatively limited in the following few weeks. However, after the number of cumulative confirmed cases exceeded 100 on February 21, 2020, the spread of the virus began to progress rapidly. On March 26, 2020, the Tokyo government officially released a policy calling for self-restriction, including closure, shortened business hours, and limited capacity in entertainment venues in the Tokyo metropolitan area. Moreover, the government declared the state of emergency to further induce self-restriction behaviors. Although the rate of new infections temporarily decreased during the implementation of the policies and the national emergency response ended on May 25, 2020, the transmission of COVID-19 continued. The number of daily new confirmed cases has been increasing since July 2020 and eventually peaked in December 2020. To mitigate and interrupt the spread of COVID-19, the Japanese government implemented the second state of emergency from January 8 to March 21, 2021, to limit individual mobility and promote social distancing. As indicated by the labels in Figure 1, the targeted period can be separated into several time windows according to these key time points.

To balance the benefits and costs of social distancing measures, the Japanese government imposed them mainly as recommendations; thus, citizens were expected to voluntarily comply by, for example, refraining from performing outdoor activities and avoiding gatherings. In this sense, because the success of social distancing behaviors depends on spontaneous public acceptance and compliance, it is imperative to investigate not only which demographic groups are more or less likely to accept and comply with the restrictions but also how the compliance changes across time. It should be noted that although social distancing measures were still accompanied by voluntary compliance without legal penalties even during the period of the state of emergency, more restrictive policies, including canceling events, closing down nonessential business facilities, and reducing business hours, were implemented to minimize physical contact. Nevertheless, as a particular case, the policy implementation's effect that primarily relies on voluntary compliance remains unclear. Particularly, because Japan experienced multiple waves of the rapid spread of COVID-19 infection and states of emergency, the impact of policy implementations might vary over time. Investigating how compliance with social distancing measures was affected by policy implementation throughout the period of the COVID-19 pandemic can provide essential insights into clarifying whether a “mild lockdown” [21] policy is the appropriate intervention strategy for guiding social distancing behaviors.

## Methods

### Study Design

This study combined several data sources, including mobile tracking data and geographical statistics, to estimate mobility dynamics in entertainment venues across demographic groups. First, we established a definition of entertainment venues based on geographical statistic data related to the constitution of the facility and the workers’ type in the specific area. After specifying entertainment venues, we used the visiting population of these entertainment venues to construct a mobility index as the proxy of social distancing behaviors. Finally, we combined the mobility data with demographic information and investigated how social distancing behaviors varied across demographic groups and shifted over time.

The key data sources and definitions are introduced next.

### Mobile Tracking Data

In this study, we used mobility data provided by DOCOMO Insight Marketing, Inc [22], 1 of the biggest telecom companies in Japan. Using the cell towers, the locations of the individuals were recorded on an hourly basis and aggregated as the estimated population with a 500 m grid cell. Particularly, based on the profile of the mobile subscribers, geolocation information was aggregated into demographic groups. Furthermore, the original data were preprocessed through extrapolating estimation. Thus, mobility population can be used to reflect the actual condition of mobility without bias in the adoption rates of NTT DOCOMO mobile terminals by age group, gender, and residential area [22,23]. Mobile tracking data provide high-spatial, longitudinal, and demographic-specific mobility populations for examining how mobility patterns vary across demographic groups over time. We focused on the mobility data recorded in the Tokyo metropolitan ﻿area from January 1 to March 31, 2021. This period covers the time before and during the COVID-19 outbreak in Japan, allowing for a comprehensive analysis of mobility behaviors over time.

### Specification of Entertainment Venues

This study focused on the mobility in entertainment venues in which most of the space was used for leisure, for example, restaurants and bars. To specify major entertainment venues in the Tokyo metropolitan area, we used granular land-use data from the Statistics Bureau of Japan (2016) that includes area-feature information on the composition of establishments and workers in 500 m grid cells by industry. In our case, we defined the industrial divisions “accommodation, eating, and drinking services” and “living-related, personal, and amusement services” as an entertainment-related category and assumed that areas where the proportion of establishments and workers was high could be considered “entertainment venues.” More specifically, the eligibility criteria of a 500 m grid cell that identified as an entertainment venue were (1) the number of entertainment category–related establishments was >100, (2) the number of entertainment category–related workers was >500, (3) the proportion of entertainment category–related establishments was >0.4, and (4) the proportion of entertainment category–related workers was >0.4.

### Mobility Population and Mobility Index

As outcome measures, this study assessed social distancing behaviors based on metrics related to the mobility population. The mobility population indicates the volume of the population tracked in entertainment venues, which could be easily computed by matching the specified 500 m grid entertainment venues and the corresponding mobility data. However, the mobility population cannot be directly applied to estimate the dynamic of social distancing behaviors, as the dynamic of the mobility population may have an inherent seasonal pattern. Instead, a mobility index was computed to reflect the extent to which mobility changed because of the COVID-19 pandemic during the study period, which we compared with the baseline. More specifically, we used the mobility population in entertainment venues in 2019 as a baseline that represents the level of the mobility population without the effect of the pandemic. Next, we compared the daily time series of the aggregated mobility population since 2020 with this baseline to compute the mobility index, which controlled potential seasonality factors that may affect mobility patterns other than those of the pandemic. Formally, for a specific demographic group “i,” distinguished by age and gender, we used the following formula to estimate the mobility index since 2020:




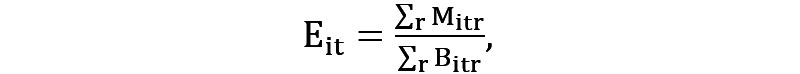




where r = {r_1_, r_2_, . . . r_n_} denotes a list of entertainment venues and “t” denotes the date of mobility population tracked. Here, 
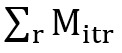
 is the mobility population in all defined entertainment venues of demographic group “i” during a specific date, and *
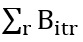
* is the mobility population of the corresponding date in 2019 as a baseline.

Beyond the macro seasonal variation, such estimated mobility index might still be biased due to the inherent variation in the mobility population during the week.

**Figure 2:**
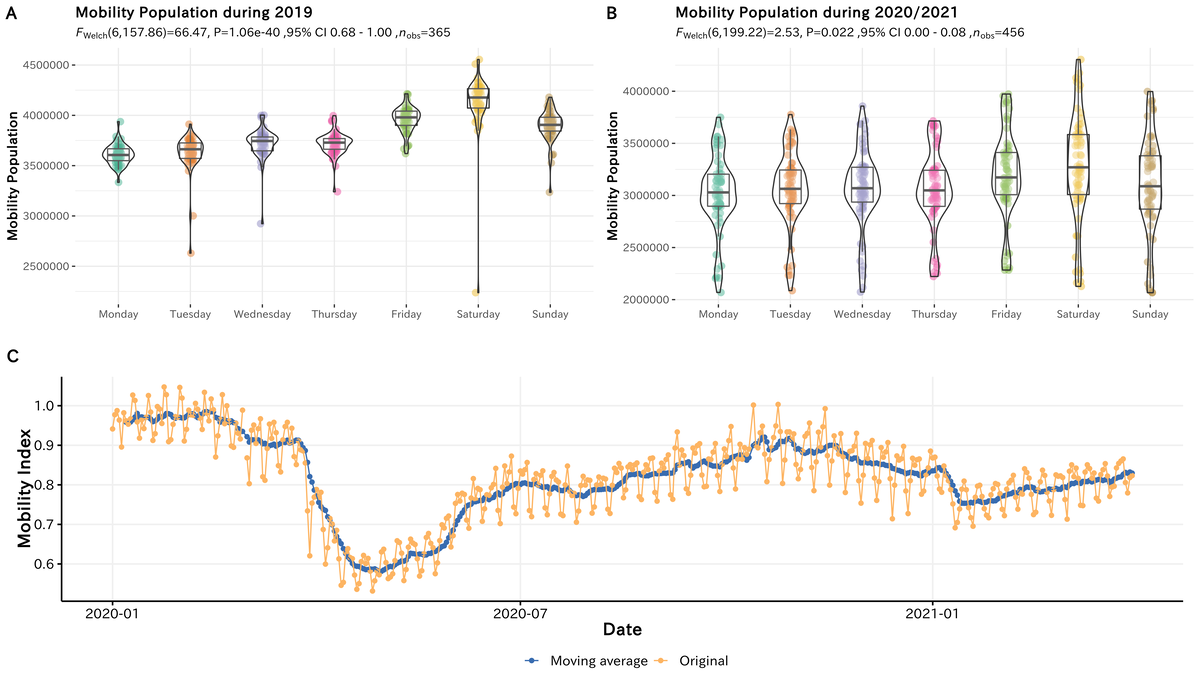
(A) Weekly variation in the mobility population during 2019. (B) Weekly variation in the mobility population since 2020. (C) Comparison of the original mobility index and the 7-day moving average of the mobility index.

As shown in Figures 2A and 2B, before the pandemic, the mobility population in

entertainment venues was high during weekends in 2019, while since 2020, the magnitude of variation decreased due to the pandemic. Since the comparison with the baseline was according to the date, for example, when the specific date is a weekday in 2020 but a weekend in 2019, the mobility index would be estimated biasedly smaller and vice versa. To exclude the day-of-the-week effects, we further processed the mobility index by computing the 7-day moving average as follows:




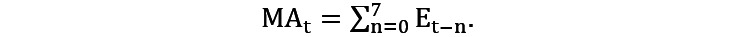




As shown in Figure 2C, the dynamic of the original mobility index seems to exhibit instability due to the day-of-the-week effects. Through the computation of the moving average, the dynamic of the estimated mobility index tends to be smooth and can still represent the overall trend of the original mobility index. As statistical analysis can be sensitive to the bias in the original mobility index, we used the moving average of the mobility index to compute social distancing behaviors in the following analysis.

The main objective of this study was to provide a comprehensive understanding of how voluntary and policy-induced social distancing behaviors shift over time across demographic groups. We conducted the analysis using 2 methods.

### Voluntary Social Distancing Behaviors

To investigate voluntary social distancing behaviors, we focused on the temporal relationship between the prevalence of infectious and the estimated mobility index of entertainment venues. We assumed that individuals’ voluntary compliance with social distancing behaviors was based on their risk perception, which is related to the recent prevalence of infectious. In practice, we used a cross-correlation function (CCF) to examine the association between time-lagged daily cases of infection in Tokyo and the moving average of the mobility index. A CCF measures chronological relations between the time series “x” and the time-shifted time series “y.” In our case, the estimated coefficient of the CCF can be interpreted as a metric that describes how recent cases of infection affected mobility in entertainment venues. In practice, we incrementally shifted the daily cases of infection back forward from 0 to 7 days and computed the CCF between the mobility index and the daily cases of infection at different lags.

### Policy-induced Social Distancing Behaviors

Beyond the social distancing behaviors induced by the risk perception, we also focused on how policy intervention induced the change in social distancing behaviors. More specifically, we focused on the 2 post-policy-intervention periods in the Tokyo metropolitan ﻿area—from March 26 to May 5, 2020, and from January 1 to March 22, 2021—and then used the Bayesian structural time series (BSTS) model [24] to dynamically investigate the policy-induced social distancing behaviors. BSTS models can simulate counterfactual trends based on the model training on the pretreatment time series, that is, predicted counterfactual series that would have occurred in a virtual counterfactual scenario with no intervention. Subsequently, the causal effect of the intervention can be determined by computing the pointwise relative impact, and the cumulative causal impact can be assessed by comparing the real postintervention observed series and the predicted postintervention observed series.

Our research design used the BSTS models to investigate how policy intervention affects the estimated mobility index in entertainment venues. In addition, BSTS models are allowed to incorporate covariates likely to affect the outcome of interest to control for spurious effects and unobservable dynamics. Notably, time-varying covariates are assumed to be unaffected by the effects of intervention treatment. In practice, we assumed that mobility in the entertainment venues was likely to be influenced by weather conditions. Thus, we incorporated the mobility index, temperature, precipitation, and wind velocity into BSTS models that integrate weather conditions to fit the trend of the mobility index.

### Analysis Framework

In summary, our analyses are organized as follows.

First, we extracted the mobility population within the entertainment venues of each demographic group to access social distancing behaviors.

Subsequently, we computed the mobility index to capture the dynamics of social distancing behaviors during the COVID-19 pandemic. On the one hand, we used the mobility population for 2019 as a baseline to compute the mobility index and to control for seasonality variation and the imbalanced population of the demographic groups. On the other hand, we computed the 7-day moving average of the mobility index to investigate the dynamics of social distancing behaviors and to control for the day-of-the-week effect.

Next, with the estimated mobility index, we used the CCF to examine the effect of the increase in infectious cases on social distancing behaviors and the degrees of this influence across the demographic groups and periods of the COVID-19 pandemic.

Moreover, we used the BSTS model to investigate the effect of the state of emergency in Japan on social distancing behaviors. Particularly, the model was computed for 2 states of emergency. In this manner, the study provided insight into changes in the policy-induced social distancing behaviors of different demographic groups during the COVID-19 pandemic in Japan.

## Results

### Validation of the Estimated Mobility Index in Entertainment Venues

**Figure 3:**
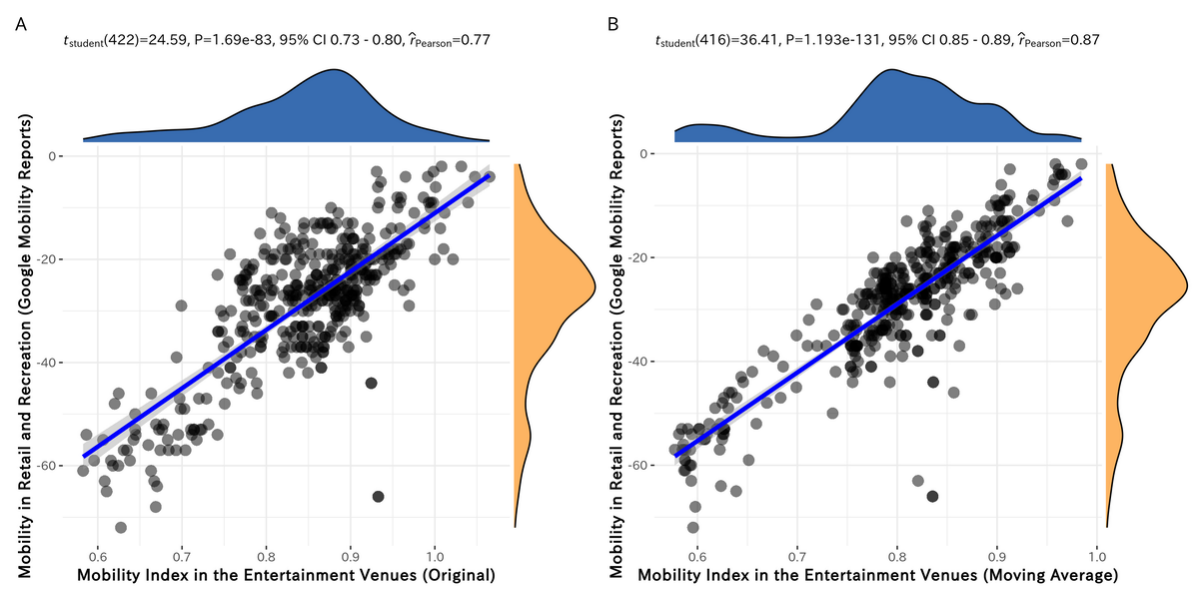
Correlation between the estimated mobility index and Google’s mobility report on retail and recreation.

As a quality control of our estimation, we cross-validated the estimated mobility index in entertainment venues by comparing it with the time series data provided by Google Mobility Reports [25], which suggest the extent to which mobility in a certain category of places changes compared with the baseline. As shown in Figure 3, we compared the mobility in the “retail and recreation” category in Tokyo and our estimated mobility index in the entertainment venues for each day. Both the original mobility index (*R*^2^=0.77, 95% CI 0.73-0.80, *P*<.001) and the moving average of the mobility index (*R*^2^=0.87, 95% CI 0.85-0.89, *P*<.001) were highly correlated with the outcome of Google Mobility Reports, indicating that our estimation of the mobility that occurred in entertainment venues was generally reasonable.

### General Mobility Dynamics by Gender and Age

This section presents the general mobility dynamics of each demographic group. Vertical dashed lines indicate the period in which the state of emergency was implemented in Tokyo metropolitan areas.

**Figure 4:**
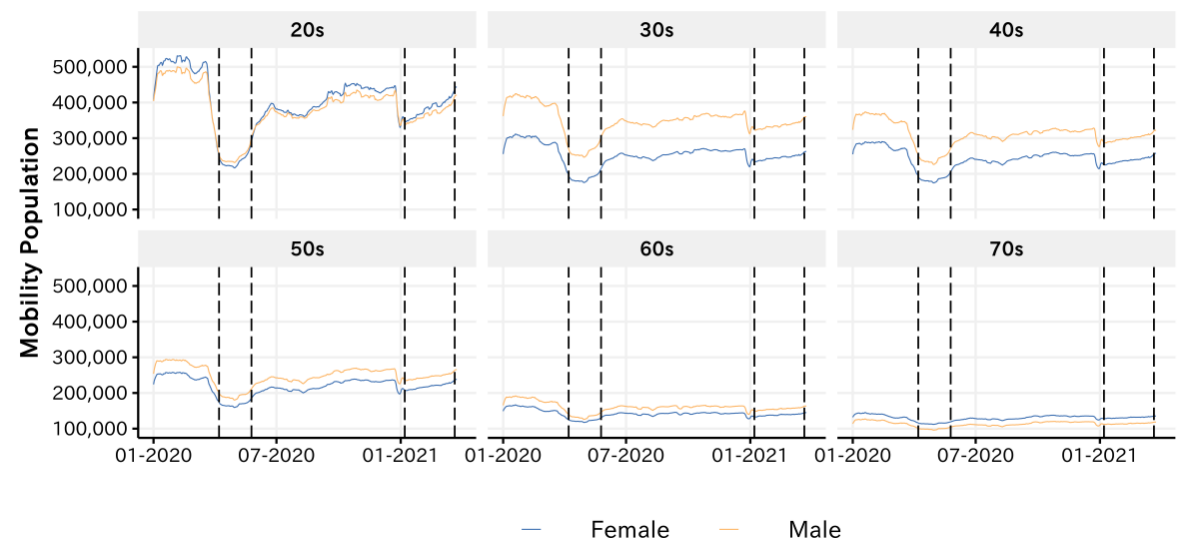
Dynamics of the mean average mobility population by age and gender.

Figure 4 presents the dynamics of the mobility population in the entertainment venues by gender and age. On the one hand, by comparing the mobility pattern from the perspective of gender, we found that the general mobility population of males is higher than that of females. Particularly, among individuals aged 30-70 years, the mobility population of males was significantly higher than that of females [8]. On the other hand, from the perspective of age, the mobility populations of individuals in their twenties were significantly higher than those of the elderly, which implies that individuals in their twenties constitute the majority of the population in entertainment venues. For details on the comparison of mobility populations among demographic groups, please refer to Multimedia Appendix 1.

**Figure 5:**
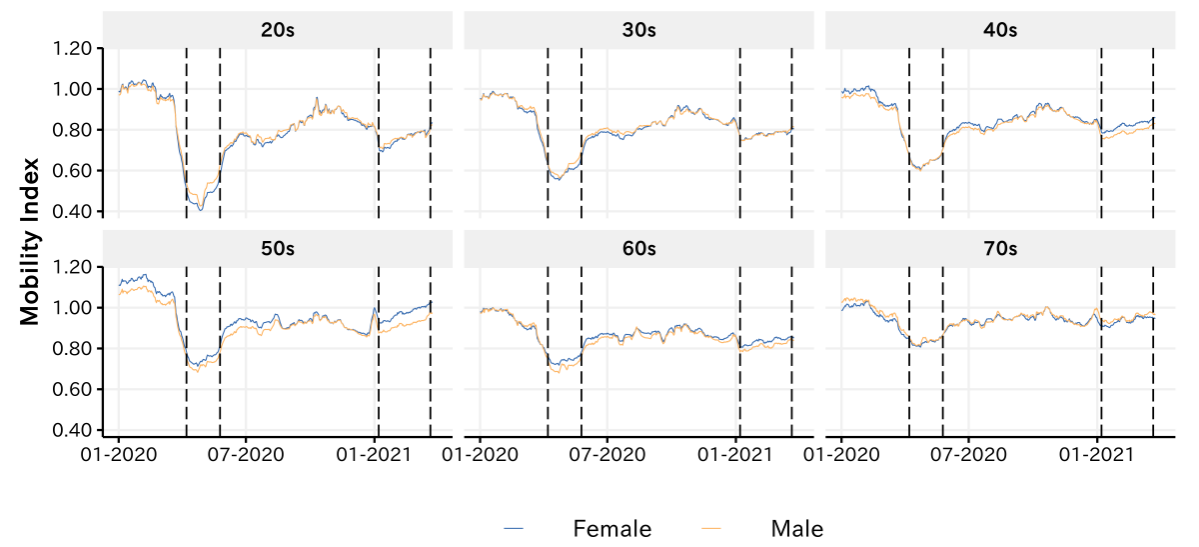
Dynamics of the mean average mobility index by age and gender.

Figure 5 presents the dynamics of the estimated mobility index in entertainment venues by gender and age. Generally, we observed a similar dynamic pattern across demographic groups: the mobility index significantly decreased across demographic groups after the outbreak of the COVID-19 pandemic in Japan. For a detailed comparison of the mobility index between the pre-COVID-19 period and the COVID-19 pandemic, please refer to Multimedia Appendix 2. Also consistent was that when the policy interventions were implemented, a significant decrease in the mobility index was observed, followed by a rebound to the pre-policy-intervention after lifting of the policy interventions. In general, no significant differences were observed in the estimated mobility index between males and females, whereas the dynamics of the estimated mobility index appeared to vary across age groups. Particularly, after the outbreak of the COVID-19 pandemic, the mobility index of individuals in their twenties appeared to decrease more significantly than that of the elderly, which implies that a large proportion of youths were adopting social distancing behaviors.

### Voluntary Social Distancing Behaviors

A typical driver of voluntary social distancing behaviors was the risk perception induced by the increasing number of infections. Here, we used the CCF to examine the association between the prevalence of infections and the dynamics of voluntary social distancing behaviors across demographic groups over time. Specifically, negative correlation coefficients indicated that the increase in the cases of infection could have led to the decrease in the mobility index in entertainment venues, which we interpreted as the magnitude of voluntary social distancing behaviors.

According to the period of the COVID-19 pandemic in Japan defined earlier, we computed the CCF for each demographic group during the different periods of the COVID-19 pandemic.

**Figure 6:**
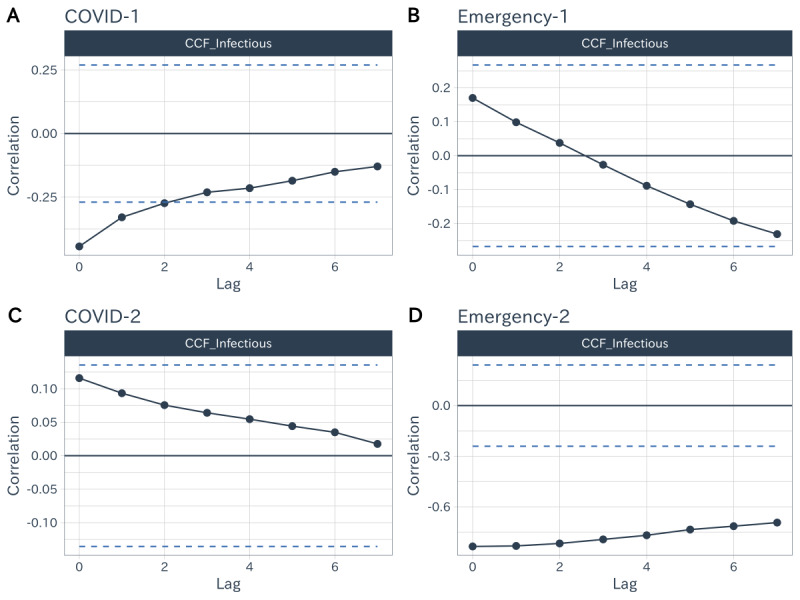
Cross-correlation between the number of cases of infection and the mobility index during the periods of the COVID-19 pandemic. CCF: cross-correlation function.

Although no systematic differences in voluntary social distancing behaviors were observed among demographic groups (please refer to Multimedia Appendix 3 for detailed analysis results), we found that the pattern of voluntary social distancing behaviors shifted during different periods of COVID-19 pandemic. Figure 6 presents the cross-correlation between the number of cases of infection and the mobility index during the different periods of the COVID-19 pandemic in Japan. In Figure 6, the horizontal dashed lines indicate the boundary of white noise, and the values of the CCF must lie beyond the interval to be significant. We observed that during the COVID-1 period, the number of infectious cases was negatively correlated with the mobility index at lags from 0 to 2 days, which indicated that the recent increasing prevalence of infections can lead to a decline in visiting entertainment venues. However, statistical significance of association was not observed during both the Emergency-1 period and the COVID-2 period. This finding indicates that the increasing number of cases of infection rarely affects visits to entertainment venues, that is, the magnitude of voluntary social distancing behaviors has gradually decreased during these periods. During the Emergency-2 period, the number of cases of infection and mobility index became strongly correlated again, indicating that the second declaration of the state of emergency significantly activated the voluntary social distancing behaviors.

### Policy-induced Social Distancing Behaviors

To examine the impact of policy intervention, we used the BSTS method to predict the counterfactual mobility dynamics in entertainment venues and could thus quantitatively analyze how policy interventions affect social distancing behaviors. More specially, we set 2 types of models to evaluate the impact of 2 policy interventions, respectively: (1) the COVID-1 period as the pretreatment period and the Emergency-1 period as the posttreatment period and (2) the COVID-2 period as the pretreatment period and the Emergency-2 period as the posttreatment period.

**Figure 7:**
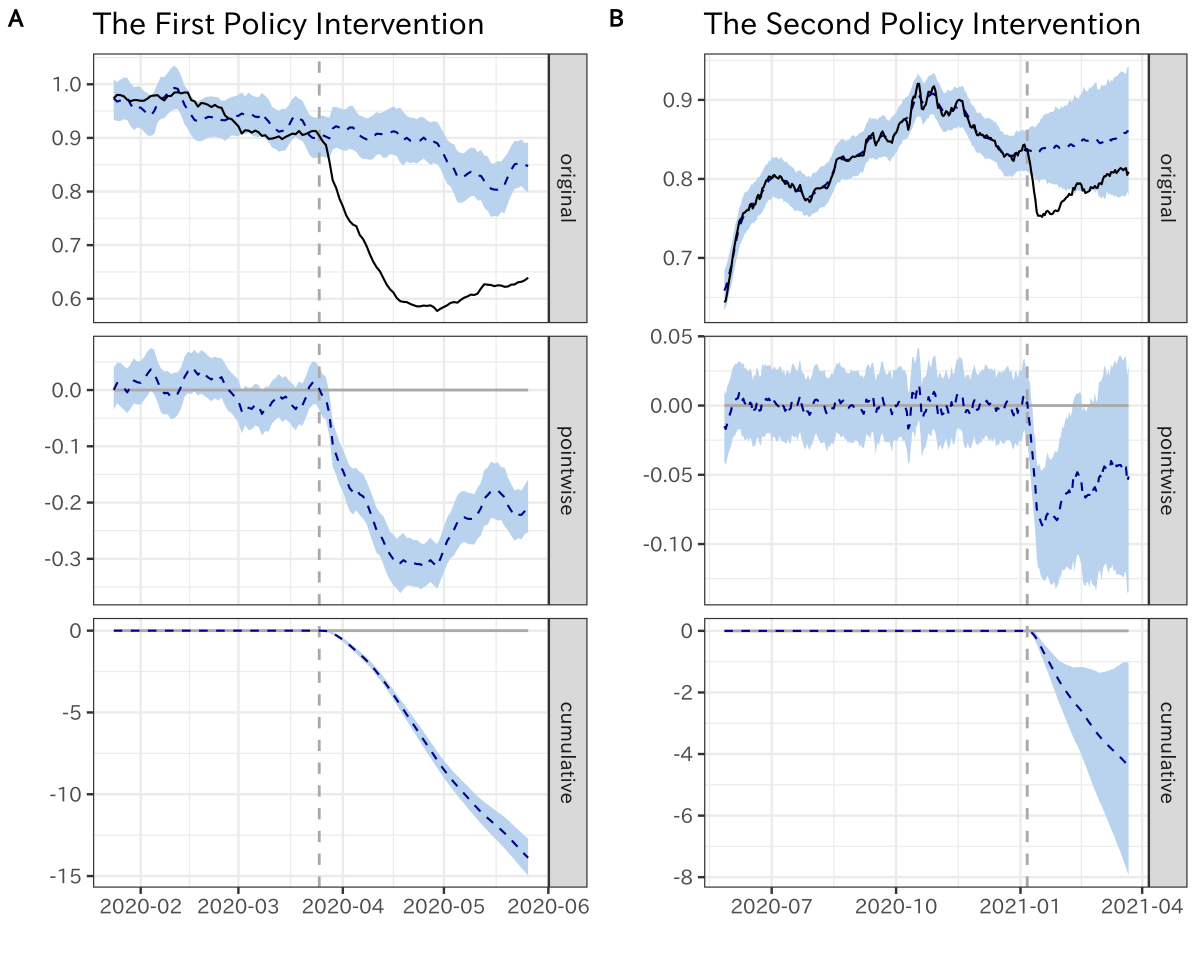
Time series of the Bayesian structural time series (BSTS) model.

Figure 7 demonstrates how the BSTS model made the predictions and constructed the counterfactual predictions for accessing the interventions’ impact. More specifically, the vertical dashed line denotes the day that the state of emergency was released, the solid series denotes the real dynamics of the mobility index, and the dashed series indicates the counterfactual trends that would have been observed without intervention.

For both computations, we found that during the pretreatment period, the BSTS model successfully captured the long-term trend and the fluctuations caused by the covariates. After the intervention, we observed considerable differences between the 2 series, which were summarized in terms of pointwise differences and cumulative differences.

**Figure 8:**
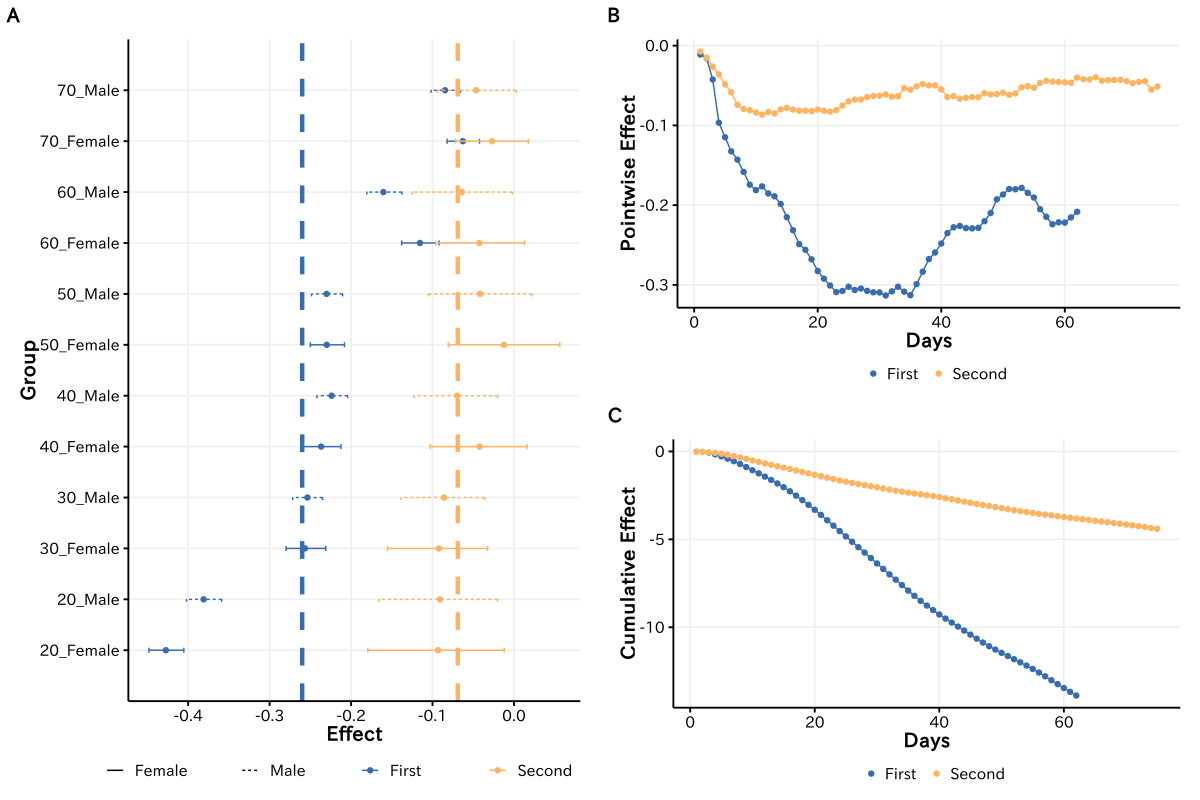
(A) Relative impact of 2 policy interventions across demographic groups. (B) Comparing the pointwise impact between the 2 policy interventions. (C) Comparing the cumulative impact between the 2 policy interventions.

In practice, we measured the impact of the 2 policy implementations on different demographic groups separately. Figure 8A presents the relative impact among different demographic groups. In Figure 8A, the vertical dashed line indicates the general impact of the 2 policy interventions that were computed on the whole population, which can serve as the baseline of policy impact.

Generally, as the estimated coefficients were lower than 0 across demographic groups, the impact of policy implementations was applicable to the whole population. However, we found that the magnitude of the effect appeared to vary by demographic groups over time.

On the one hand, for the first policy intervention, we observed a considerable decrease in the mobility index among most demographic groups. Especially, the mobility index for females aged 20-29 years decreased by 42.73% and that of males aged 20-29 years decreased by 40.19%, which is significantly higher than elderly individuals. These findings indicate that the policy interventions had the most significant impact on youths.

On the other hand, for the second policy intervention, although it also caused the visiting population to entertainment venues to slightly decrease, the magnitude of the impact substantially dropped compared to the former policy intervention among all demographic groups. That is, although the state of emergency was announced again to restrict nonessential outdoor activity, many people maintained their regular behavior pattern rather than complying with the social distancing requests, and the impact of such policy implementation largely declined.

Beyond the general magnitude of the impact, because the length of the 2 policy intervention periods was similar, we compared and investigated how the dynamic pattern of impacts varied between the 2 policy interventions.

We present Figures 8B and 8C to compare the dynamics of the pointwise impact and the cumulative impact, respectively, between the 2 policy interventions. For both 2 policy interventions, we observed that the implementation of the policy interventions consistently led to a decrease in visits to entertainment venues during the initial few days, while the impact of policy interventions decreased in the following days. In other words, the policy-induced compliance with the social distancing measures tended to have a transient phase—with a significant reduction in mobility for the initial policy implantation—and the impact of the policy on compliance willingness substantially decreased over time. Here, we specifically compared transient trends between the 2 policy interventions. On the one hand, as shown in Figure 7B, the time point that the impact turned to decrease was much earlier for the second policy intervention than for the first policy intervention. On the other hand, as shown in Figure 7C, the gradient of the cumulative impact was much higher for the first policy intervention than for the second policy intervention. In summary, these comparisons suggest that the impact of the first policy intervention on social distancing behaviors was not only significantly greater but also tended to be much longer-lasting compared to that of the second policy intervention.

## Discussion

### Principal Findings

In this study, we used mobility data to investigate how social distancing behaviors vary among demographic groups. The disparity and dynamics of social distancing behaviors driven from our analysis can have critical implications for optimal disease control policy design and implementation.

First, our findings demonstrate distinct patterns of social distancing behaviors and their dynamics across age groups. More specifically, we found that the population in entertainment venues comprised mainly individuals aged 20-40 years, which implies that this age group could be exposed to a higher risk of infection in entertainment venues. However, the larger amount of population did not necessarily suggest that the individuals in this age group are more likely to violate the restrictions. On the one hand, based on the dynamics of the estimated mobility index, among the age groups, the extent of reduction in the frequency of visiting entertainment venues during the pandemic was generally higher among younger individuals, particularly individuals aged 20-30 years. On the other hand, by investigating the impact of policy interventions, the rates of acceptance and compliance with the social distancing policy interventions were also higher among younger individuals. From this perspective, the increasing contribution of the youth to the spread of infection should be more likely attributed to their size instead of their refusal to observe social distancing behaviors. Indeed, the adoption of social distancing behaviors is less likely induced by policy implementations among the elderly. As the COVID-19 pandemic progresses, increased nuanced and targeted responses are required to effectively control the prevalence of the infection. Given the age-dependent disparity, governments should tailor mobility restrictions to the targeted population. Especially, the existing policy implementations are seemingly inefficient for the elderly, who are more susceptible to the severe symptoms of and mortality posed by COVID-19. Thus, target mitigation strategies might be necessary to increase the intention of elderly individuals to adopt mobility restriction behaviors.

Second, our analysis also provides insights into the dynamics of voluntary social distancing behaviors over time. Our proposed mechanism assumes the connection between voluntary social distancing behaviors and the increasing number of infections mediated by risk perception. Accordingly, we propose that the dynamics of voluntary social distancing behaviors can be explained by the dynamic of risk perceptions. Our results indicate that in the initial phase of the COVID-19 pandemic, the increasing number of cases of infection could have led to the decrease in entertainment venue visiting; thus, voluntary social distancing behaviors have resulted in important outcomes in terms of reducing unnecessary physical contact during this period. However, with the progress of the pandemic, the significance of such association declined, which indicates that the risk perceptions about COVID-19 have decreased over time. This scenario may be attributed to the decreased adherence of the public to social distancing measures and vigilance toward COVID-19 [26]. Moreover, we find that policy interventions can strengthen risk perceptions. Specifically, although voluntary social distancing behaviors largely diminished during the COVID-2 period or the second state of emergency, policy intervention appeared to increase the awareness of the severity of the pandemic and concerns regarding COVID-19, leading to an increase in voluntary social distancing behaviors. In this sense, policymakers should continue to alert the public about the risk of COVID-19 in order to promote voluntary social distancing behaviors.

Third, our results indicate the importance of implementing the public health policy promptly to limit the spread of the COVID-19 infection. Quantifying the impact of policy interventions is crucial for policymaking. Here, 2 insights deserve emphasis. On the one hand, although the social distancing interventions in Japan were less strict than those in some other countries, they still significantly promoted social distancing behaviors under the implementation of a state of emergency, which is in with previous investigations [27], although the adoption of social distancing behaviors resurged and then gradually resumed to the normal level after lifting the policy interventions. On the other hand, our results warn policymakers that the effectiveness and impact of self-restriction recommendations appeared to decrease in response to the second wave of COVID-19. Particularly, in the second state of emergency in Japan, the magnitude of the reduction in the visiting flow to entertainment venues was limited compared to the first state of emergency. Furthermore, the initial, strong impacts could only last for a short time and could quickly enter the decreasing phase.

### Limitations

The findings of this study should be carefully considered in the context of its 2 main limitations.

The first limitation is that we focused on the mobility flow in entertainment venues as a proxy to estimate social distancing behaviors; however, visiting entertainment venues is only 1 aspect related to compliance with social distancing measures. Nevertheless, this study presented an example of how to integrate aggregated mobility data with geological statistics data. Hence, we suggest that in further research, the proposed analysis framework that integrated mobility data and geographical statistics data be applied to monitor other aspects of social distancing behaviors. This research direction would be promising in attempts to extend the methodology to specify other types of locations (eg, residential areas or business districts); subsequently, mobility data could be used to investigate social distancing behaviors from the perspective of compliance with stay-at-home orders or remote work orders. Another direction for further research would be to provide a more comprehensive set of insights into social distancing behaviors.

The second limitation is that the implication related to policy-induced social distancing behaviors is based on the scenario of the policy implementation in Japan, where social distancing measures were accomplished through spontaneous cooperation. Thus, caution should be exercised when interpreting the implications, because it may not be applicable to other regions or countries, where the enforcement of social distancing was stricter than that in Japan.

### Conclusion

Given the costs of the enforced policy, many countries have decreased the stringency of the containment policy. Thus, voluntary social distancing behaviors are expected to play a critical role in future responses to the COVID-19 pandemic, even for countries that mainly relied on the enforced containment policy at the initial phase. From this perspective, implications derived from Japan could be generalized to other countries and serve as guidance for the effective induction of voluntary social distancing behaviors to combat the long-term COVID-19 pandemic.

## Appendix

Multimedia Appendix 1 Comparison of mobility population across demographic groups.

Multimedia Appendix 2 Comparison of mobility index before and during the COVID-19 pandemic.

Multimedia Appendix 3 Cross-correlation across demographic groups and time periods.

## Abbreviations

CCF: cross-correlation function
BSTS: Bayesian structural time series
RQ: research question
