# Supplementary material for: The Disparity and Dynamics of Social Distancing Behaviors in Japan: Investigation of Mobile Phone Mobility Data
Source: JMIR Med Inform. 2022 Mar 22;10(3):e31557. doi: 10.2196/31557 (PMC8942095; doi:10.2196/31557)
Supplement: Multimedia Appendix 1 [file medinform_v10i3e31557_app1.docx]

**Appendix**

**Comparison of mobility population across demographic groups**


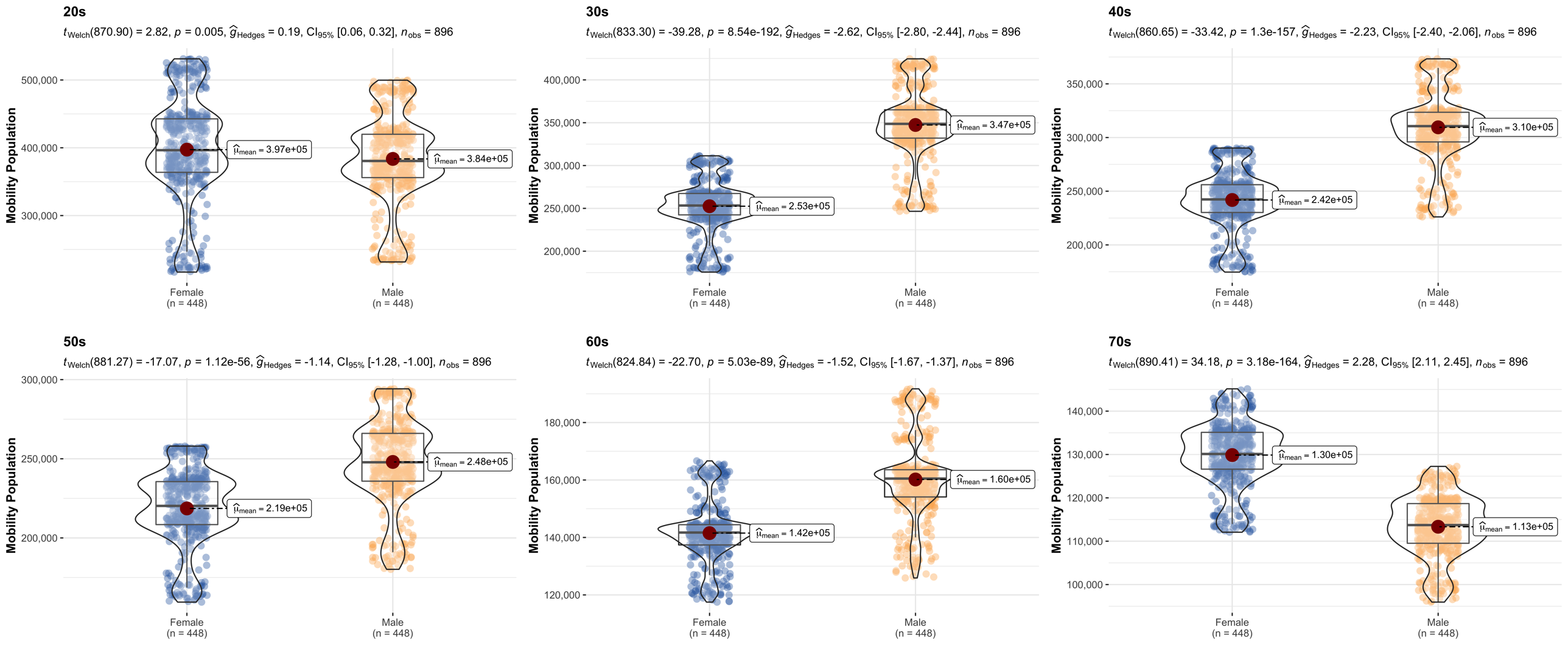


**Figure A.1. Comparison of mobility population among gender groups.**

Each subplot in Figure A.1 presents the comparison of daily mobility population between males and females for each age group. Intuitively, among individuals aged 30 to 70 years, the mobility population of males was significantly higher than that of females.


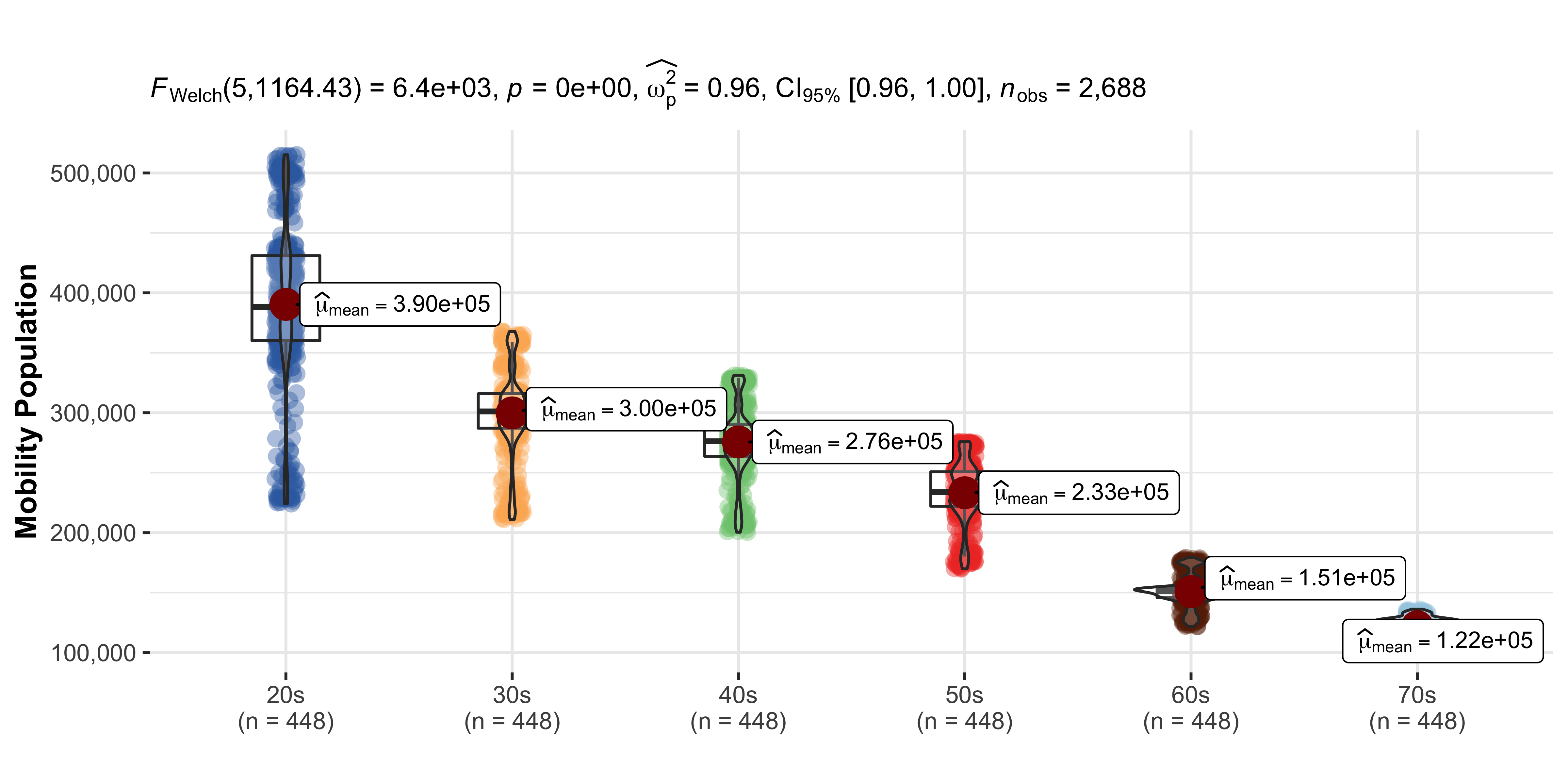


**FigureA.2. Comparison of mobility population among age groups.**

Figure A.2 demonstrates the comparison of the daily mobility population across age groups. Intuitively, youth aged 20–29 years compose the majority of the mobility population in entertainment venues. In addition, the size of the mobility population decreases with the increase in age.
