# Supplementary material for: The Disparity and Dynamics of Social Distancing Behaviors in Japan: Investigation of Mobile Phone Mobility Data
Source: JMIR Med Inform. 2022 Mar 22;10(3):e31557. doi: 10.2196/31557 (PMC8942095; doi:10.2196/31557)
Supplement: Multimedia Appendix 2 [file medinform_v10i3e31557_app2.docx]

**Appendix**

**Comparison of mobility index before and during the COVID-19 pandemic**


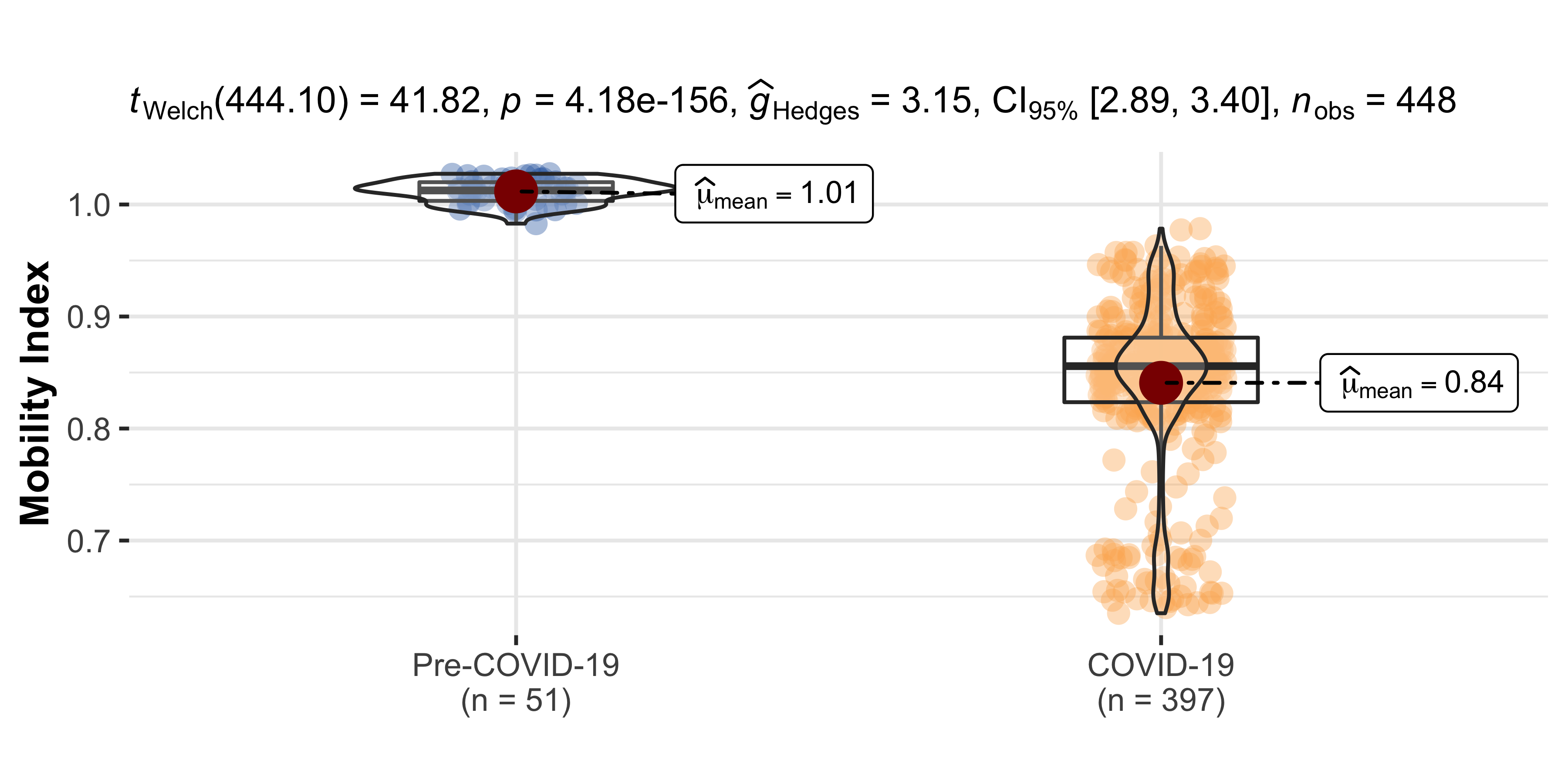


**Figure A.3. Comparison of mobility index between pre-COVID-19 period and COVID-19 pandemic**

Figure A.3 compares the daily estimated mobility index before and during the COVID-19 pandemic. Specifically, we define the pre-COVID-19 period as ranging from January 1, 2020 to February 20, 2020, during which COVID-19 was less prevalent in the Tokyo metropolitan areas. On February 21, 2020, the cumulative confirmed cases exceeded 100, whereas the spread of the virus began to progress rapidly since then, which can be considered the beginning of the COVID-19 outbreak in Tokyo metropolitan areas. We find that the mean of the mobility index during pre-COVID-19 period is roughly 1, which implies that the mobility population remained the same compared with corresponding periods in 2019. On the contrary, the mobility index significantly decreased during the COVID-19 pandemic, which suggests that the pandemic led to considerable changes in mobility population.
