# Supplementary material for: The Disparity and Dynamics of Social Distancing Behaviors in Japan: Investigation of Mobile Phone Mobility Data
Source: JMIR Med Inform. 2022 Mar 22;10(3):e31557. doi: 10.2196/31557 (PMC8942095; doi:10.2196/31557)
Supplement: Multimedia Appendix 3 [file medinform_v10i3e31557_app3.docx]

**Appendix**

**Cross-correlation across demographic groups and time periods**


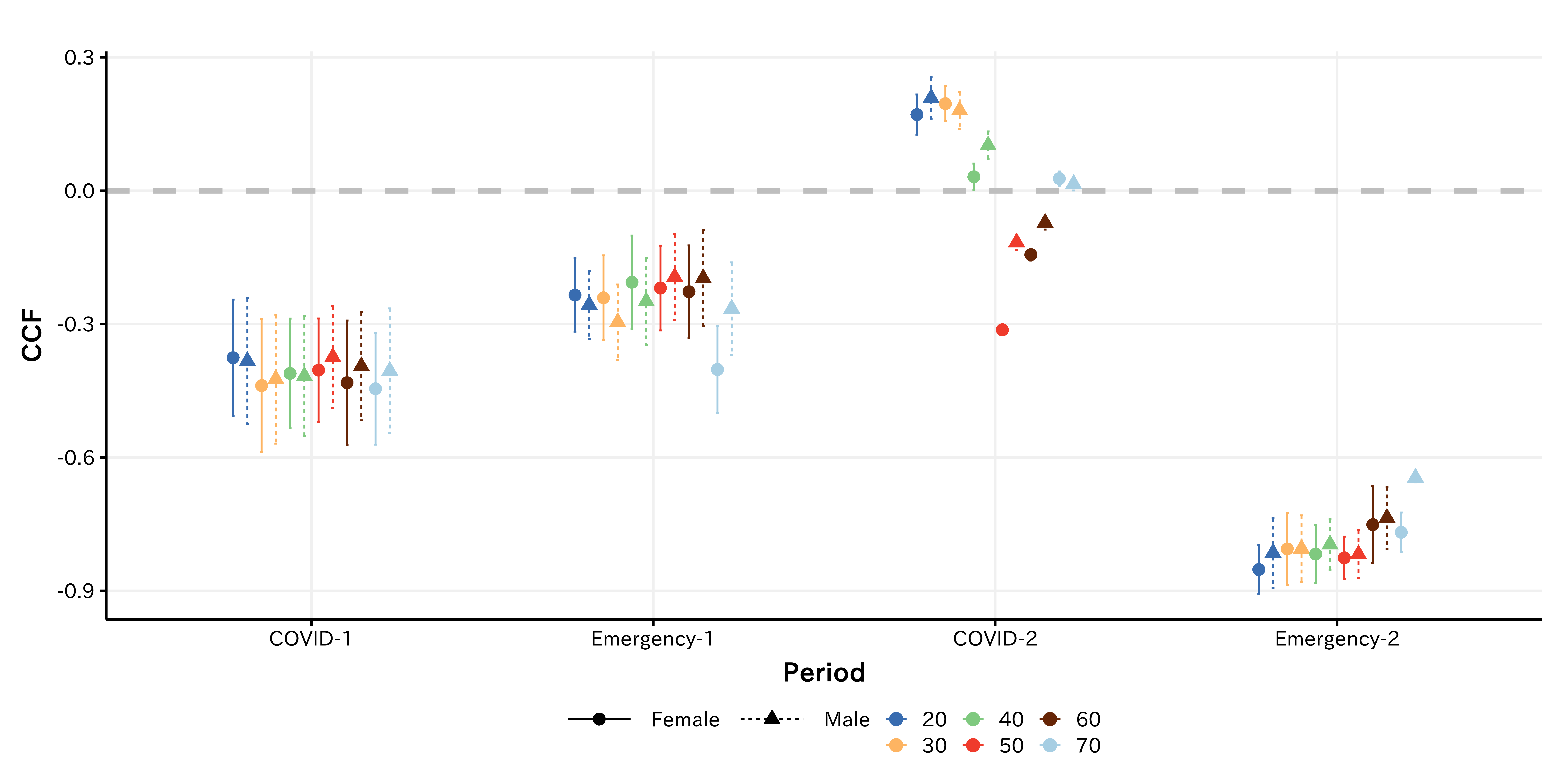


**Figure A.4. Cross-correlation across demographic groups and time periods**

In Figure A.4, we computed the CCF for each demographic group across time periods. However, we observed no systematic differences in voluntary social distancing behaviors among the demographic groups
